# Development of DNA Microarray for Parallel Detection of Community-Acquired Pneumonia Bacterial Pathogens

**DOI:** 10.17691/stm2024.16.2.02

**Published:** 2024-04-27

**Authors:** N.A. Sakharnov, E.N. Filatova, M.I. Popkova, S.L. Slavin, O.V. Utkin

**Affiliations:** PhD, Senior Researcher, Laboratory of Molecular Biology and Biotechnology; Academician I.N. Blokhina Nizhny Novgorod Scientific Research Institute of Epidemiology and Microbiology of Rospotrebnadzor (Russian Federal Consumer Rights Protection and Human Health Control Service), 71 Malaya Yamskaya St., Nizhny Novgorod, 603950, Russia; PhD, Leading Researcher, Laboratory of Molecular Biology and Biotechnology; Academician I.N. Blokhina Nizhny Novgorod Scientific Research Institute of Epidemiology and Microbiology of Rospotrebnadzor (Russian Federal Consumer Rights Protection and Human Health Control Service), 71 Malaya Yamskaya St., Nizhny Novgorod, 603950, Russia; MD, PhD, Leading Researcher, Laboratory of Molecular Biology and Biotechnology; Academician I.N. Blokhina Nizhny Novgorod Scientific Research Institute of Epidemiology and Microbiology of Rospotrebnadzor (Russian Federal Consumer Rights Protection and Human Health Control Service), 71 Malaya Yamskaya St., Nizhny Novgorod, 603950, Russia; Student; National Research Lobachevsky State University of Nizhny Novgorod, 23 Prospekt Gagarina, Nizhny Novgorod, 603950, Russia; PhD, Head of the Laboratory of Molecular Biology and Biotechnology; Academician I.N. Blokhina Nizhny Novgorod Scientific Research Institute of Epidemiology and Microbiology of Rospotrebnadzor (Russian Federal Consumer Rights Protection and Human Health Control Service), 71 Malaya Yamskaya St., Nizhny Novgorod, 603950, Russia

**Keywords:** DNA microarray, detection of bacterial pathogens, community-acquired pneumonia, *S. pneumonia*, *H. influenzae*

## Abstract

**The aim of the study** was to develop an experimental version of a DNA microarray for parallel detection of community-acquired pneumonia bacterial pathogens.

**Materials and Methods:**

We studied the samples of the pharyngeal mucosa smears taken from children aged 1–15 years with X-ray confirmed pneumonia. The selection of DNA probes for specific detection of community-acquired pneumonia pathogens (*S. pneumoniae*, *H. influenzae*, *M. pneumoniae*, *C. pneumonia*, and *L. pneumophila*) and development of the microarray design were carried out using the disprose program. The nucleotide sequences of pathogens were obtained from NCBI Nucleotide database. In the research we used CustomArray microarrays (USA). For a pooled sample containing *S. pneumoniae* and *H. influenzae* DNA, we performed a sequential selection of the best combinations of hybridization parameters: DNA fragment size, DNA amount, hybridization temperature. The selection criteria were: the percentage of effective probes with a standardized hybridization signal (SHS) ≥3 *Z*, and the excess of SHS levels of effective specific probes compared to SHS of effective nonspecific probes. We selected the probes to detect of *S. pneumoniae* and *H. influenzae* characterized by an effective hybridization signal under optimal conditions. The developed microarray was tested under the selected conditions on clinical samples containing *S. pneumoniae* or *H. influenzae* DNA. Using ROC analysis there were established threshold values for the signals of specific probes at optimal sensitivity points and the test specificity, the excess of which was interpreted as the evidence of pathogen presence in a sample.

**Results:**

A microarray design included 142 DNA probes to detect *S. pneumoniae*, *H. influenzae*, *M. pneumoniae*, *C. pneumoniae*, and *L. pneumophila*, the probes being synthesized onto slides. Using the example of clinical samples containing *S. pneumoniae* and/or *H. influenza* DNA, we selected optimal parameters for DNA hybridization on microarrays, which enabled to identify bacterial pathogens of community-acquired pneumonia with sufficient efficiency, specificity and reproducibility: the amount of hybridized DNA was 2 μg, the DNA fragment size: 300 nt, hybridization temperature: 47°C. There was selected a list of probes for specific detection of *S. pneumoniae* and *H. influenzae* characterized by an effective hybridization signal under the identified conditions. We determined the threshold values of standardized probe signals for specific detection of *S. pneumoniae* (4.5 *Z*) and *H. influenzae* (4.9 *Z*) in clinical samples.

**Conclusion:**

A DNA microarray was developed and synthesized for parallel indication of bacterial pathogens of community-acquired pneumonia. There were selected the optimal parameters for DNA hybridization on a microarray to identify bacterial pathogens — *S. pneumoniae* and *H. influenzae*, and determined the threshold values of significant probe signals for their specific detection. The interpretation of the microarray hybridization results corresponds to those obtained by PCR. The microarray can be used to improve laboratory diagnostics of community-acquired pneumonia pathogens.

## Introduction

Community-acquired pneumonia (CAP) is an acute infectious inflammatory process of the pulmonary tissue occurring in community (outside the hospital) or diagnosed within the first 48 h after admission, and accompanied by low respiratory infection signs, as well as radiographic findings [1].

Community-acquired pneumonia is one of the most common infectious pathologies worldwide. According to European Respiratory Society, in the countries of European Community the total number of CAP patients annually exceeds 3 million people. Every year in the USA 5–6 million of CAP cases are recorded; among them over a million of cases require hospitalization [2]. CAP rate in Europe and North America accounts for 5–10 cases per 1,000 of population [3]. According to WHO, in 2019 CAP mortality was 2.6 million cases that ranked the forth leading mortality cause in the world [4].

In Russia CAP also makes significant contribution to the infectious incidence structure. The long-term annual average being 391.82 cases per 100,000 of population in 2022, CAP incidence was 407.29 cases per 100,000 of population [5]. CAP mortality in Russia is 17–18 cases per 100,000 of population, it depending on severity and patients’ individual characteristics (age, comorbidity, the immune system condition) [6].

CAP agents include many bacterial pathogens, the main one is *Streptococcus pneumonia* causing up to 30–50% of cases. *Haemophilus influenza* in community setting occurs approximately in 10% cases, both in Russia and worldwide. It is known, that 8–30% cases of non-severe CAP are known to be caused by *Chlamydophila pneumonia* and *Mycoplasma pneumonia* [6].

Despite the large-scale implementation of moleculargenetic and other advanced techniques into laboratory practice, the proportion of diagnosed CAP of this etiology reaches only 40–60% [7].

Timely identification of CAP agent is significant to make a right choice when managing patients, and determining anti-epidemic measures to take [8–13]. However, CAP symptoms are characterized by variability and non-specificity, and for this reason the development of new methods to detect etiological CAP agents using modern technologies still continues to be relevant.

The development of DNA microarrays enabling to detect in parallel a wide range of CAP agents can be very promising to solve the problem. Many of the currently developed DNA microarrays aim at typing the pathogens associated with respiratory diseases. So, the staff members of Engelhardt Institute of Molecular Biology, Russian Academy of Sciences (Russia), developed gel DNA microarrays of low density for typing *Mycobacterium tuberculosis* strains and identifying genetic determinants of drug resistance [14]. In addition, in cooperation with D.I. Ivanovskiy Institute of Virology (Russia), they developed DNA microarray for typing influenza A virus used in order to monitor epidemiologically the agent circulation [15]. In Russia, previously, there was carried out no development of DNA microarrays aimed at detecting a wide spectrum of respiratory agents.

In 2019 powered by Agilent (USA) there was developed a DNA microarray for serotyping *S. pneumoniae*, its accuracy being compatible with PCR technique [16].

Microarrays by Affymetrix (USA) [17] and Genomica (Spain) [18] have gained wide spread, and made it possible to identify a wide range of bacterial pathogens. One of the last developments is a DNA microarray of low density described by Ma et al. [19], the microarray is aimed for determining 15 types of CAP-associated bacteria.

However, a wide range of pathogens, which are detected by microarrays described before, determine high cost and sample preparation complexity, since a significant number of specific primers are needed for tests that hampers the application of microarrays in the laboratory practice. Another disadvantage of these microarrays is impossibility to differentiate carrier state and pathogen-associated infection [19]. In such situation there would be urgent to develop DNA microarrays characterized by an optimal relationship of research cost, productivity and the finding accuracy. This problem can be solved using DNA microarrays of high density, enabling to use random primers in sample preparation, preserving the assay sensitivity and specificity. Moreover, the quality of the results obtained will also depend significantly on using optimal protocols of sample preparation and the favorable ratio of material hybridization parameters [20]. The parameters include the size of fragments and the amount of hybridized DNA, hybridization temperature, and others.

**The aim of the study** was to develop an experimental version of a DNA microarray for parallel detection of community-acquired pneumonia bacterial pathogens.

## Materials and Methods

### Development of DNA microarray design and its synthesis

We previously set forward the algorithm of selecting DNA probes for specific detection of CAP agents, it being implemented in the form of the disprose program (DIScrimination PRObe SElection) coded in program language R [21]. Using the algorithm there was developed the DNA microarray design for identifying the main bacterial CAP agents circulating in the world: *Streptococcus pneumoniae*, *Haemophilus influenzae*, *Mycoplasma pneumoniae*, *Chlamydophila pneumoniae*, *Legionella pneumophila.* We adjusted target and nonspecific bases of nucleotide sequences used when selecting DNA probes with the help of this algorithm, from the database NCBI Nucleotide [22]. The probe distribution scheme of a slide surface was drawn out using the application — Layout Designer (CustomArray, USA). The probes were synthesized on slides CustomArray Blank Slide 12K (CustomArray, USA). There were synthesized on slides the probes of negative control (NC — specific to *Rhizobium rubi* genome selected using the mentioned algorithm), and the probes of quality control (QC), nonspecific to the sequences of the pathogens under study, which were established by the platform manufacturer (CustomArray, USA). The probes were synthesized using a modified amidophosphite technique on B3 Synthesizer (CustomArray, USA) according to the manufacturer protocol [23, 24] and using a reagent kit (Sigma-Aldrich, USA, Germany, France; Panreac, Spain; Merck Sharp & Dohme, USA; Biochim, Russia).

### Materials

We studied the samples of the pharyngeal mucosa smears taken from children aged 1–15 years with X-ray confirmed pneumonia, they were under in-patient treatment in Nizhny Novgorod medical facilities (Russia).

All the presenters of under-legal-age patients gave their informed consent in accordance with Declaration of Helsinki (2013).

### Sample collection for research

The presence of CAP agent DNA was confirmed by PCR using the kit — Gen Pak DNA PCR test (Galart-Diagnosticum, Russia) to detect *S. pneumoniae*, *H. influenza*, and *L. pneumophila*, as well as the kit “AmpliSense® Mycoplasma pneumoniae/Chlamydophila pneumoniae-FL” (Central Research Institute of Epidemiology of Rospotrebnadzor, Russia) to detect *M. pneumonia* and *C. pneumoniae* DNA. In order to choose the parameters of DNA hybridization on a microarray and assess the reproducibility of results, we pooled 18 samples containing *S. pneumonia* DNA (18/18, 100% samples) and/or *H. influenza* DNA (9/18, 50% samples). To test the detectability of *S. pneumonia* and *H. influenza* in clinical samples, we used three samples of each containing *S. pneumonia* or *H. influenza* alone. A pool of six samples of oral swabs from healthy donors containing none DNA of CAP bacterial agents served as negative samples.

### DNA sample preparation and its hybridization on a microarray

We purified DNA from the samples using a kit of reagents RIBO-prep (Central Research Institute of Epidemiology of Rospotrebnadzor, Russia). We additionally cleaned DNA using 3 M sodium acetate (pH 7.0) and isopropanol (Biochim, Russia). DNA, in amounts of 2–3 μg, was fragmented by a kit of reagents NEBNext dsDNA Fragmentase (New England Biolabs, Great Britain). DNA concentration was measured spectrophotometrically using Eppendorf Bio Photometer Plus (Germany). Fragmented DNA was concentrated with isopropanol in the presence of 3 M sodium acetate (pH 7.0). The amplification of DNA (1.2 μg) was performed using a reagent kit Encyclo Plus PCR kit (Evrogen, Russia) and random decanucleotide primers Random (dN)10-primer (Evrogen, Russia) using an amplifier MaxyGene Gradient (Axygen, USA), the reaction temperature profile being the following: denaturation at 95°C — 1 min, 30 cycles (95°C — 15 s, 30°C — 1 min, 72°C — 45 s), the final elongation — 8 min. The obtained DNA was concentrated by cooled isopropanol at the presence of 3 M sodium acetate (pH 7.0). DNA (in the amount of up to 4 μg) was used as a matrix for *in vitro* replication by a reagent kit “DNA-polymerase I E. coli (Klenov fragment)” (SibEnzyme, Russia) and random decanucleotide primers Random (dN)10-primer (Evrogen, Russia). Biotin labeling was inserted into synthesized DNA by replacing a half of deoxyuridine triphosphate (dUTP) with its biotinylated analogue Bio-12-dUTP (DNA-synthesis, Russia). The obtained biotin-labeled DNA was concentrated by cooling with isopropanol in the presence of 3 M sodium acetate (pH 7.0). The resulting amount of biotin-labeled DNA varied within the range of 2.0–3.5 μg, which was enough for DNA hybridization on a microarray. The target biotin-labeled DNA were hybridized on a microarray followed by washing (for microarray reusing) according to the manufacturer’s instruction (CustomArray, USA). There was determined an optimal combination of three hybridization parameters: target DNA fragment size (200, 300, 400 nt), the amount of hybridized DNA (1, 2, 3 μg) and hybridization temperature (40, 42, 45, 47, 50°C).

### Mathematical processing and analysis of hybridization signals

Hybridization signals in the form of ECD files were exported into CSV format using Electra Sense Analysis v. 3.4.2 (CustomArray, USA). The calculations were made in freely distributable programming support environment R v. 3.6.1 [25].

Primary hybridization signals were standardized and received a standardized hybridization signal (SHS) expressed by the formula:

Z=(X−MNC)/SDNC,

where *X* — a primary hybridization signal of the probe understudy, M_NC_ — arithmetic mean of the signals from negative control probes, SD_NC_ — standard deviation of the signals from negative control probes.

The probes with SHS over 10 *Z* were assessed as those with nonspecific/partial binding, and were excluded from the following analysis. The signal level over 3 *Z* was estimated as effective [26], over 4 *Z* — high, over 5 *Z* — very high.

For every hybridization parameters combination we determined hybridization quality indicator: effectiveness, specificity — the relationship of specific and nonspecific signals, as well as the relationship of sensitivity and specificity parameters. We tested each of the parameters combination sequentially selecting the best variants.

Hybridization efficiency was assessed as the percentage of effective probes from the total test probes. To assess the effect the hybridization parameters have on the ratio of specific and nonspecific signals, we calculated the median relationship of SHS specific and nonspecific probes. As specific there were used those designed for identifying *S. pneumonia* and *H. influenzae*, and as nonspecific — the probes to identify *M. pneumoniae*, *C. pneumoniae*, *L. pneumophila.* ROC-analysis was used to determine the sensitivity-specificity ratio.

### ROC-analysis

In carrying out ROC-analysis we calculated the microarray sensitivity and specificity when selecting significant signal threshold (SST). By SST we meant such SHS probe level, the excess of which made it possible to interpret the resulting hybridization as the evidence of the agent presence in a DNA sample. When calculating, only effective probes were taken into consideration. Sensitivity and specificity were determined for each SST under testing according to the following formulas:

Sensitivity = TP / (TP + FN);Specificity = TN / (TN + FP),

where TP — the number of true-positive probes (the probes designed for detecting *S. pneumonia* and *H. influenzae*, their standardized signal being over the threshold level under test); FP — the number of false positive probes (the probes designed for detecting *M. pneumoniae*, *C. pneumonia*, and *L. pneumophila*, their standardized signal being over the threshold level under test); TN — the number of true-negative probes (the probes designed to detect *M. pneumoniae*, *C. pneumonia*, and *L. pneumophila*, their standardized signal being lower or equal to the threshold level under test); FN — the number of false-negative probes (the probes designed to detect *S. pneumonia* and *H. influenzae*, their standardized signal being lower or equal to the threshold level under test).

Based on the obtained values, we plotted a ROC-curve, calculated the area under curve (AUC), determined SST corresponding to maximum Youden’s index (the optimal sensitivity and specificity relationship), and SST corresponding to maximum test specificity.

### Results reproducibility assessment

A pooled DNA sample was hybridized on three different slides after single hybridization and the following washing, as well as three times on one slide with the following washing. Spearman’s rank correlation coefficient was calculated to determine the correlation between electrochemical signals of hybridization slides, as well as we calculated the coefficient of variation (*Cv*) of every probe on different slides according to the formula:

Cv=(SD/M)⋅100%,

where SD — the standard deviation of SHS probe, M — arithmetic mean of probe SHS on all tested slides.

### Selection of effective probes for detecting S. pneumonia and H. influenzae

The sequences of specific probes designed for detecting *S. pneumonia* and *H. influenzae*, and demonstrating effective hybridization in selected parameters of the pooled sample hybridization, were aligned in relation to the reference sequence of the appropriate pathogen genome. As a reference for *S. pneumonia* we used the sequence “*Streptococcus pneumonia* R6, complete sequence” (in database NCBI Nucleotide — NC_003098), for *H. influenzae* — the sequence “*Haemophilus influenza* strain Hi375 chromosome, complete genome” (in database NCBI Nucleotide — NZ_CP009610).

Locally, the nucleotide sequences were aligned using the program BLASTN of the program package BLAST+ v. 2.10.0 [27]. There were determined the areas of reference genome, which the selected probes were aligned on, the identity being 100% in the absence of point discordance and nucleotide loss. For annotation of the revealed reference genome we used NCBI Nucleotide data base [22]. Then in order to broaden the panel of the used probes, there were chosen all probes (making the microarray design and specific to the revealed regions), which were designed to detect *S. pneumonia* or *H. influenzae*.

### Microarray testing

The developed DNA microarray was tested for CAP agents (*S. pneumonia* and *H. influenza*) in clinical samples. An effective signal from a pool of specific probes selected at the previous stage was considered as a specific signal. Using ROC-analysis we determined SST corresponding to maximum test specificity. SST as calculated separately for *S. pneumonia* and *H. influenzae*.

### Data statistical processing

The data were analyzed in freely distributable software R v. 3.6.1. We used statistical approaches determining arithmetic mean (M), standard deviation (SD), median (Me), first and third quartiles (Q1 and Q3), Spearman’s correlation coefficient (ρ), coefficient of variation (*Cv*) with ROC curve and determining AUC and Youden’s index.

## Results

### Probe sequence choice for detecting community acquired pneumonia DNA

Using the disprose program we selected the probes for specific detection of five bacterial CAP agents: *S. pneumoniae*, *H. influenzae*, *M. pneumoniae*, *C. pneumoniae*, *L. pneumophila*. A pool of candidate probes was formed by separating reference genome sequences into the regions of stated length. Candidate probes were selected by physicochemical parameters: probe length (24–32 nt), nucleotide (guanine, cytosine) percentage composition (40–60%), the number of homogenous repetitions (less than five similar nucleotides in succession), minimal folding energy (≥0 kcal/mol), melting temperature (55–60°C). For testing the capability of candidate probes to hybridize with target sequences, we aligned them by BLAST algorithm with target and non-specific sequence bases. Then using the disprose program we selected maximum specific probes according to the following parameter: those identical to target sequences (covering — 100%, no point discordance and gaps), and no cross hybridization with non-target sequences (covering — less than 50%). As a result, we selected by 30 probes to detect *S. pneumoniae*, *M. pneumoniae*, *C. pneumoniae*, *L. pneumophila*, and 22 probes — to detect *H. influenzae* (see Appendix). The selected probes were successfully synthesized on slides Blank Slide 12K (CustomArray, USA). Each slide consisted of 4 identical sectors, each of them having 142 target probes, 90 negative control probes, and 90 synthesis quality control probes.

### The effect of hybridization parameters on hybridization quality characteristics

We sequentially selected the best combinations of hybridization parameters: DNA fragment size (200, 300, 400 nt), DNA amount (1, 2, 3 μg), and hybridization temperature (40, 42, 45, 47, 50°C) — for a pooled sample containing *S. pneumonia* and *H. influenzae* DNA. When hybridizing a negative sample, no signals of effective probes were detected on all tested parameters.

**Figure 1.**
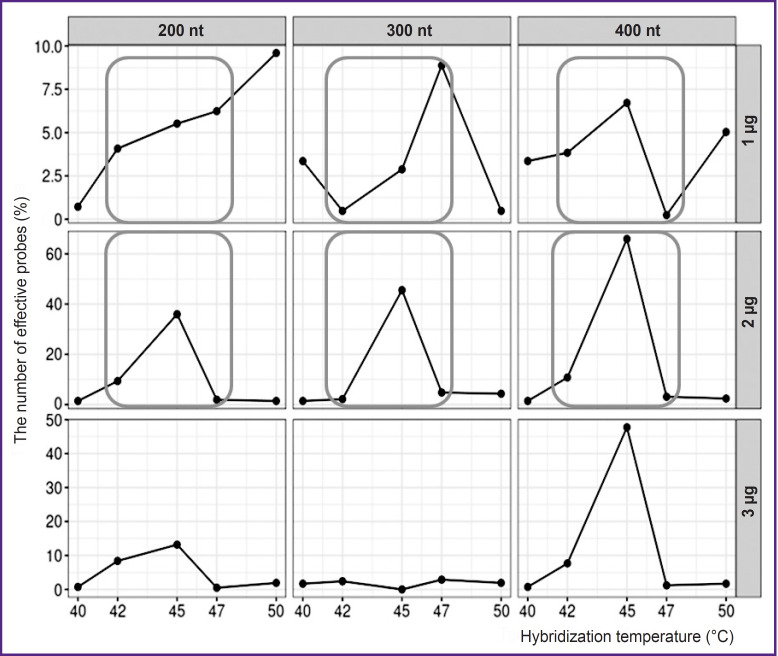
Dependence of probe hybridization efficiency on hybridization parameters 200, 300, 400 nt — length of hybridized DNA fragments; 1, 2, 3 μg — amount of hybridized DNA; the parameters selected for further optimization are framed

The maximum hybridization effectiveness was revealed in the temperature range 42–47°C. Hybridization efficiency decreased when DNA amount lowered from 2 to 1 μg, as well as in increasing the amount up to 3 μg. No dependence of hybridization efficiency on DNA fragment length was found (Figure 1).

**Figure 2.**
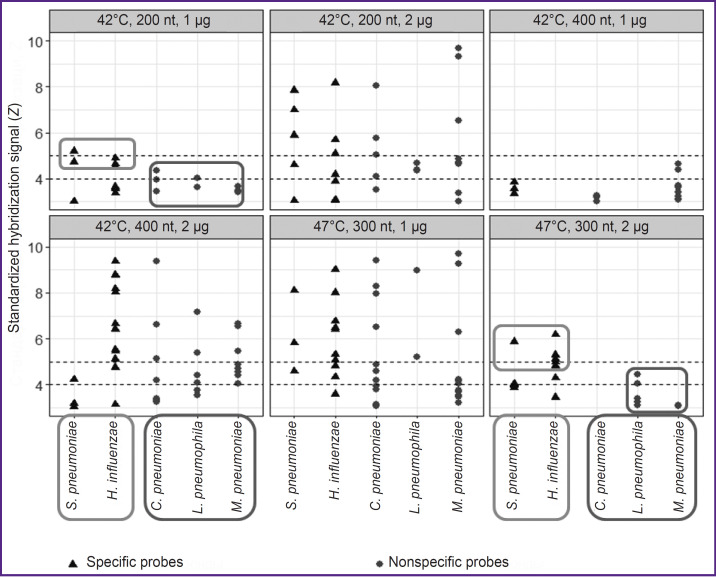
Standardized hybridization signal values of effective specific and non-specific probes in different hybridization parameters 42, 47°C — hybridization temperature; 200, 300, 400 nt — the length of hybridized DNA fragments; 1–2 μg — the amount of hybridized DNA. Dotted line indicates the borders of high level signal 4 *Z* and very high level signal — 5 *Z*. Frames mark the parameters selected for further optimization

Hybridization specificity analysis showed the most effective specific probes (to detect *S. pneumonia* and *H. influenzae*) and the highest signal of specific hybridization were in the following combination of parameters: 1) DNA, 1 and 2 μg, 200 and 400 nt, 42°C; 2) DNA, 1 and 2 μg, 300 nt, 47°C. However, there were also revealed the signals of effective nonspecific probes (for *M. pneumoniae*, *C. pneumonia*, and *L. pneumophila*) (Figure 2).

The resulting selection revealed two combinations of hybridization parameters, which provided the maximal number of effective specific probes. The medians of their levels exceeded those of effective signals from nonspecific probes:

DNA, 1 μg, 200 nt, 42°C — 9 specific probes with SHS=4.5 [3.6; 4.7] *Z* and 9 non-specific probes with SHS=3.6 [3.5; 3.9] *Z*, 1.3 times exceeding;

DNA, 2 μg, 300 nt, 47°C — 13 specific probes with SHS=4.3 [4.0; 5.1] *Z*, and 8 nonspecific probes with SHS=3.2 [3.1; 3.6] *Z*, 1.3 times exceeding (see Figure 2).

ROC analysis findings showed the first combination of hybridization parameters (DNA, 1 μg, 200 nt, 42°C) AUC ROC-curve value to be 0.57, while the second combination (DNA, 2 μg, 300 nt, 47°C) — 0.89. The second variant was chosen as optimal hybridization parameters; for this combination the maximum Youden’s index corresponded to SST=3.5 *Z* (specificity — 0.75; sensitivity — 0.85) and SST=4.5 *Z* (specificity — 1.00; sensitivity — 0.46) corresponded to maximum specificity.

Thus, optimal hybridization parameters are DNA, 2 μg, 300 nt, 47°C.

### Reproducibility assessment

In hybridization under selected conditions, the microarrays were characterized by high reproducibility of signals received on different slides, and successively on one slide. Correlation coefficient ρ was 0.93 [0.92; 0.94], and coefficient of variation *Cv* — 9.3 [7.2; 11.3]%.

### Characteristics of the probes specifically detecting S. pneumonia and H. influenzae

Based on the data on pooled sample hybridization at optimal parameters from those presented in microarray design, for further application there were chosen the most effective probes to detect *S. pneumoniae* and *H. influenzae* (Table 1). For *S. pneumoniae* detection we chose 6 from 30 specific probes presenting on a microarray; 5 from 6 probes were specific to *S. pneumoniae* genome regions encoding or complementary coding proteins, and 1 probe was specific to non-annotated genome area. For *H. influenzae* detection we chose all 22 from 22 specific probes presenting on a microarray. 14 among them were complementary to non-annotated of *H. influenzae* genome area; and 8 — to two regions, which encode proteins.

**T a b l e 1:** Characteristics of the probes specifically detecting *S. pneumoniae* and *H. influenza*

Probe number	Length (nt)	Probe sequence	Genome region coordinates*, complimentary probe sequence (nt)	Genome region characteristics
str_1436936	28	agagttcctcctcttcataagtctatcc	218389–218416	Unannotated genome region
str_836861	26	gacattataggacgtactgagcatac	224989–225014	Coding gene region *pflF*, gene product — formate acetyltransferase 3 (AAK99036, NCBI Protein)
str_1768809	30	gcaacaaagcaagtagactagacagaacaa	1363254–1363283	Genome region, complementary to gene encoding area, gene product — ABC transporter (AAL00185, NCBI Protein)
str_1768807	30	aagcaacaaagcaagtagactagacagaac	1363252–1363281	
str_344828	24	gtctcctgtaacgccaaagacatt	1828896–1828919	Genome region, complementary to gene encoding area *ackA*, gene product — acetate kinase (AAL00657, NCBI Protein)
str_166887	24	ctgtttaaacccgaagaaggagtt	896865–896888	Encoding region of gene *phtE*, gene product — pneumococcal histidine triad Е precursor (AAK99712, NCBI Protein)
hi_100185	24	catcaatgaaatgaagccctgtcg	591988–592016	Non-annotated genome region
hi_100186	24	atcaatgaaatgaagccctgtcga	591989–592017	
hi_100187	24	tcaatgaaatgaagccctgtcgat	591990–592018	
hi_100188	24	caatgaaatgaagccctgtcgatt	591991–592019	
hi_416136	25	acatcaatgaaatgaagccctgtcg	591987–592016	
hi_416137	25	catcaatgaaatgaagccctgtcga	591988–592017	
hi_416138	25	atcaatgaaatgaagccctgtcgat	591989–592018	
hi_416139	25	tcaatgaaatgaagccctgtcgatt	591990–592019	
hi_711905	26	aacatcaatgaaatgaagccctgtcg	591991–592016	
hi_711906	26	acatcaatgaaatgaagccctgtcga	591992–592017	
hi_711907	26	catcaatgaaatgaagccctgtcgat	591992–592018	
hi_924234	27	aacatcaatgaaatgaagccctgtcga	59991–592017	
hi_924235	27	acatcaatgaaatgaagccctgtcgat	591992–592018	
hi_924236	27	catcaatgaaatgaagccctgtcgatt	591993–592019	
hi_458134	25	acaagtttcgtttctggggattatg	818127–818151	Locus NF38_04170 — region hypothetically encoding protein (AIT67430, NCBI Protein)
hi_458135	25	caagtttcgtttctggggattatgt	818128–818152	
hi_699475	26	aggatcaatactgttatcagagtcgc	487919–487944	Locus NF38_02410 — region hypothetically encoding protein RNA-polymerase sigma factor RpoD (AIT67106, NCBI Protein)
hi_699476	26	ggatcaatactgttatcagagtcgct	487920–487945	
hi_911551	27	caggatcaatactgttatcagagtcgc	487918–487944	
hi_911552	27	aggatcaatactgttatcagagtcgct	487919–487945	
hi_911553	27	ggatcaatactgttatcagagtcgctt	487920–487946	
hi_911554	27	gatcaatactgttatcagagtcgcttg	487921–487947	

^*^ for probe selection there were used genome sequences of data bases NCBI Nucleotide: NC_003098 *Streptococcus pneumonia* R6, complete sequence (accessed October 1, 2022); NZ_CP009610 *Haemophilus influenzae* strain Hi375 chromosome, complete genome (accessed May 3, 2023).

### S. pneumoniae and H. influenzae detection in clinical samples

When hybridizing genetic material of clinical samples of patients with CAP and the stated *S. pneumoniae* agent, among effective probes there were those specific to all five pathogens under study (*S. pneumoniae*, *H. influenzae*, *M. pneumoniae*, *C. pneumonia*, *L. pneumophila*), though the probes specific to *S. pneumoniae* DNA demonstrated higher SHS (Table 2).

**T a b l e 2:** Main hybridization characteristics of clinical samples containing *S. pneumonia* and *H. influenza*

Characteristics	Slide number_number of washings		
***Hybridization of the sample containing S. pneumoniae***			
	Slide 81559_04	Slide 81562_02*	Slide 81562_03
The number of effective probes (SHS ≥3)	16	22	14
The number of effective probes specifiic to *S. pneumoniae*	5	6	5
AUC value	1	0.59	0.74
SST of maximal point of Youden’s index*	4.30 (se=1.00; sp=1.00)	3,90 (se=0.83; sp=0.63)	3.90 (se=0.83; sp=0.94)
Minimum SST at maximum specificity point*	4.30 (se=1.00; sp=1.00)	4.50 (se=0.33; sp=1.00)	4.40 (se=0.67; sp=1.00)
Effective SHS values of specific probes, Me [Q1; Q3]:			
*S. pneumoniae*	4.91 [4.68; 5.20]	4.33 [4.03; 5.04]	4.82 [4.63; 5.76]
*H. influenzae*	3.24 [3.17; 3.71]	3.83 [3.76; 3.97]	3.15 (1 probe)
*M. pneumoniae*	4.20 [4.01; 4.20]	3.69 [3.48; 4.12]	3.87 [3.48; 4.09]
*C. pneumoniae*	3.29 [3.16; 3.34]	3.77 [3.46; 3.79]	3.53 (1 probe)
*L. pneumophila*	3.56 (1 probe)	4.13 [4.04; 4.27]	3.53 [3.35; 3.62]
***Hybridization of the sample containing H. influenzae***			
	Slide 81562_04	Slide 81563_01	Slide 81563_02
The number of effective probes (SHS ≥3)	15	15	12
The number of effective probes specific to *H. influenzae*	6	5	6
AUC value	0.93	0.88	0.93
SST of maximal point of Youden’s index*	4.90 (se=083; sp=1.00)	4.70 (se=0.83; sp=1.00)	4.00 (se=0.83; sp=0.90)
Minimal SST in maximum specificity point*	5.00 (se=0.83; sp=1.00)	4.70 (se=0.83; sp=1.00)	4.80 (se=0.33; sp=1.00)
Effective SHS values of specific probes, Me [Q1; Q3]:			
*S. pneumoniae*	3.78 (1 probe)	3.44 (1 probe)	No probes
*H. influenzae*	5.11 [5.04; 5.13]	5.20 [5.12; 5.63]	4.12 [4.04; 4.88]
*M. pneumoniae*	4.49 [4.24; 4.72]	4.04 [3.99; 4.09]	3.87 [3.61; 4.20]
*C. pneumoniae*	3.83 [3.71; 3.95]	No probes	No probes
*L. pneumophila*	3.17; 3.68 (2 probes)	3.53 [3.33; 3.81]	3.91 (1 probe)

^*^ in brackets the values of sensitivity (se) and specificity (sp) are given. SHS — standardized hybridization signal, SST — significant signal threshold.

Due to the fact that both specific and some non-specific probes were characterized by an effective hybridization signal, as SST we chose the maximum specificity point, which achieved a signal of specific probes alone. Such SST provided 100% specificity, though resulting in false-negative signals (the signals of effective specific probes interpreted as negative). SST of specific probes for *S. pneumoniae* was 4.5 *Z*. Similarly, in hybridization of clinical samples of patients with CAP and stated *H. influenzae* agent, among effective probes there were specific and nonspecific ones. In addition, the probes specific to *H. influenza* DNA were characterized by higher SHS. SST selected for the probes designed for *H. influenzae* detection was 4.9 *Z* (see Table 2).

## Discussion

In order to develop DNA microarray for parallel detection of bacterial CAP agents using our own disprose program and data base NCBI we selected the following sequences of DNA probes detecting *S. pneumoniae*, *H. influenzae*, *M. pneumoniae*, *C. pneumonia*, and *L. pneumophila*, which were successfully synthesized in a microarray. In addition to individual physic-chemical characteristics of the probes, which were determined when developing microarray design, the significant parameters, which have an effect on effectiveness and specificity of probes binding to a target DNA, are hybridization temperature and the amount of. The hybridization temperature increase and hybridizing DNA amount decrease result in an increase in binding specificity, however, it leads to efficiency decrease, and vice versa [20].

Using pooled samples of *S. pneumonia* and *H. influenzae* DNA, according to the algorithm we developed, there were successively studied various combinations of DNA hybridization parameters on a microarray: DNA fragment size, DNA amount, and hybridization temperature. By sequential sampling, we chose an optimal combination of hybridization parameters, when there were revealed the maximum effective and specific signals of probes to detect *S. pneumonia* and *H. influenzae*, namely: DNA, 2 μg, 300 nt, 47°C.

At such hybridization parameters combination, the characteristics of reproducibility of signals received both on different microarrays and sequentially corresponded to the quality characteristics specified in literature — correlation coefficient over 0.90 and correlation of variation under 15% [28]. Testing of negative pooled sample showed no effective probe signals.

The study findings demonstrate the impossibility to choose one “optimal” probe, it is necessary to assess hybridization signal of a pool of specific probes to detect each pathogen. There were selected the probes synthesized on a microarray to detect *S. pneumonia* and *H. influenza* characterized by an effective hybridization signal under optimal conditions, and specificity to the selected regions of reference genomes. There were selected 6 probes for *S. pneumonia* detection, and 22 probes — for *H. influenzae* detection (see Table 1). It should be noted that significant amount of probes for *H. influenzae* detection were specific to limited genome regions. It can be explained by the fact that the initial probe selection for *H. influenzae* detection was complicated by high genetic similarity of the pathogen with closely related agents of *H. parainfluenzae* and *H. haemolyticus*, which can be present in normoflora [29, 30]. To decrease the risk of cross probe hybridization and false-positive results, we excluded from an initial pool of candidates the sequences similar to *H. parainfluenzae* and *H. haemolyticus* genome. The rest probes were complimentary to several unique *H. influenza* genome regions. The probes for *S. pneumoniae* detection were chosen from the sequences specific to the regions located along the entire pathogen genome length.

When testing clinical samples, we stated both specific and some non-specific to be characterized by an effective hybridization signal; however, SHS levels of specific probes were significantly higher. The fact that there were revealed effective nonspecific signals when analyzing clinical samples using a developed microarray can be the evidence of possible carrier state of detected agents. According to literature data [31], bacterial agents of CAP can be a part of normal flora. So, in healthy children aged 0–6 were found to have *S. pyogenes*, *S. pneumoniae*, *H. influenza*, and *Moraxella catarrhalis* DNA, and the frequency of their revealing was comparable to the frequency of acute respiratory infections. The number of *M. pneumoniae* carrier state in healthy children varied from 3 to 58% depending on season [32], while the frequency of asymptomatic infection by *Chlamydophila pneumoniae* in children and adults was 1–6% [33, 34]. *Legionella species* carrier state remains unproved [35].

For safe detection of CAP agents DNA using a microarray, in possible carrier state, as well as in possible nonspecific hybridization of microarray probes, it is of great importance to calculate carefully the threshold values of significant hybridization signal to detect each pathogen at clinically significant concentration. The research [36] showed that there is no direct dependence between the hybridization signal levels of different probes and the concentration of detected molecules in the studied sample, and even the probes specific to one region of target sequence differ in affinity, and therefore, they differ in hybridization signal. Accordingly, SST calculation is needed for each of CAP agents under study. As SST there were chosen the points of maximum specificity (4.5 *Z* — for *S. pneumoniae*, 4.9 *Z* — for *H. influenzae*) that enabled to interpret the microarray hybridization findings in accordance with PCR results.

Thus, the developed DNA microarray in optimal hybridization parameters and significant threshold values of probe signals provide parallel detection of CAP agents DNA (*S. pneumoniae* and *H. influenzae*) with sufficient efficiency, specificity and reproducibility. The microarray is unique in Russia, and exhibits a number of advantages compared to those developed abroad. Detection of each pathogen is provided by a composition of unique specific probes that enhances the detection probability of a pathogen in a sample. Due to the use of random decanucleotide primers there have been provided the unification of biomaterial sample preparation, analysis cost reduction, and labor cost decrease. One more advantage of the developed microarray is its reusability (minimum five times without the results quality loss). The suggested algorithm of analyzing hybridization findings using signal threshold values enables to differentiate clinically significant infection and carrier state of bacterial CAP agents. The algorithm with the help of our DNA microarray can be applied to develop detection techniques for other bacterial CAP agents such as *M. pneumoniae*, *C. pneumoniae*, and *L. pneumophila*.

## Conclusion

There was developed and synthesized an experimental DNA microarray for parallel detection of community acquired pneumonia bacterial agents: *S. pneumoniae*, *H. influenzae*, *M. pneumoniae*, *C. pneumonia*, and *L. pneumophila.* In the course of the present research by an example of bacterial agents of *S. pneumonia* and *H. influenzae* there were selected optimal conditions for DNA hybridization (amount — 2 μg, fragment size — 300 nt, hybridization temperature — 47°C), which enable to get signals with sufficient specificity and reproducibility. We revealed a pool of probes for specific detection of *S. pneumoniae* (n=6) and *H. influenzae* (n=22) characterized by an effective hybridization signal in the revealed conditions. There were determined threshold values of significant signals of the probes specific detection of *S. pneumonia* and *H. influenzae* in clinical samples, which enable to interpret hybridization results (4.5 *Z* — to detect *S. pneumoniae*, and 4.9 *Z* — to detect *H. influenzae*).

A developed microarray can be used to improve laboratory diagnosis and monitoring of bacterial agents of community-acquired pneumonia.

## References

[1] Chuchalin A.G. Sinopalnikov A.I. Kozlov R.S. Avdeev S.N. Tyurin I.E. Rudnov V.A. Ratchina S.A. Fesenko O.V. Clinical guidelines on diagnosis, treatment and prophylaxis of severe community-acquired pneumonia in adults. Kliniceskaa mikrobiologia i antimikrobnaa himioterapia 2015; 17(2):84–126.

[2] Ezhlova E.B. Demina Yu.V. Efimov E.I. Brusnigina N.F. Maleev V.V. Tartakovskij I.S. Bilicheko T.N. Shkarin V.V. Kovalishena O.V. Chubukova O.A. Blagonravova A.S. Vnebol’nichnye pnevmonii: klassifikatsiya, patogenez, etiologiya, epidemiologiya, laboratornaya diagnostika na sovremennom etape. Analiticheskiy obzor [Community-acquired pneumonia: classification, pathogenesis, etiology, epidemiology, laboratory diagnostics at the present stage. Analytical review]. Moscow; 2013.

[3] Rozenbaum M.H. Pechivanoglou P. van der Werf T.S. Lo-Ten-Foe J.R. Postma M.J. Hak E. The role of Streptococcus pneumonia in community-acquired pneumonia among adults in Europe: a meta-analysis. Eur J Clin Microbiol Infect Dis 2013; 32(3):305–316, 10.1007/s10096-012-1778-4 23242464

[4] World Health Organization. The top 10 causes of death. URL: https://www.who.int/news-room/fact-sheets/detail/the-top-10-causes-of-death.

[5] Federal’naya sluzhba po nadzoru v sfere zashchity prav potrebiteley i blagopoluchiya cheloveka. Gosudarstvennyy doklad “O sostoyanii sanitarno-epidemiologicheskogo blagopoluchiya naseleniya v Rossiyskoy Federatsii v 2022 godu” [Federal Service for Supervision of Consumer Rights Protection and Human Welfare. State report: “On the state of sanitary and epidemiological well-being of the population in the Russian Federation in 2022”]. 2023. URL: https://www.rospotrebnadzor.ru/documents/details.php?ELEMENT_ID=25076.

[6] Zajcev A.A. Sinopal’nikov A.I. Practical recommendations for the management of patients with nonsevere community-acquired pneumonia. Russkij medicinskij zhurnal 2020; 4: 19–23.

[7] Zyryanov S.K. Chenkurov M.S. Ivzhits M.A. Batechko Yu.A. Ivanova E.B. Yakunina M.A. Etiology of community-acquired pneumonia and prevalence of comorbidities in elderly patient population. Klinicheskaya mikrobiologiya i antimikrobnaya khimioterapiya 2020; 22(3):242–248, 10.36488/cmac.2020.3.242-248

[8] Zaripova A.Z. Valieva R.I. Bayazitova L.T. Tselischeva M.V. Diagnosis of pneumococcal infections of the respiratory tract. Prakticheskaya pul’monologiya 2018; 4: 74–80.

[9] Bonten M.J.M. Huijts S.M. Bolkenbaas M. Webber C. Patterson S. Gault S. van Werkhoven C.H. van Deursen A.M.M. Sanders E.A.M. Verheij T.J.M. Patton M. McDonough A. Moradoghli-Haftvani A. Smith H. Mellelieu T. Pride M.W. Crowther G. Schmoele-Thoma B. Scott D.A. Jansen K.U. Lobatto R. Oosterman B. Visser N. Caspers E. Smorenburg A. Emini E.A. Gruber W.C. Grobbee D.E. Polysaccharide conjugate vaccine against pneumococcal pneumonia in adults. The N Engl J Med 2015; 372: 1114–1125, 10.1056/nejmoa1408544 25785969

[10] Falkenhorst G. Remschmidt C. Harder T. Wichmann O. Glodny S. Hummers-Pradier E. Ledig T. Bogdan C. Background paper to the updated pneumococcal vaccination recommendation for older adults in Germany. Bundesgesundheitsblatt Gesundheitsforschung Gesundheitsschutz 2016; 59(12): 1623–1657, 10.1007/s00103-016-2466-9 27885449

[11] Haritonov M.A. Zhurkin M.A. Ivanov V.V. Clinical and diagnostic features of community-acquired viral-bacterial pneumonia. Prakticheskaya pul’monologiya 2016; 1: 30–35.

[12] Aftaeva L.N. Melnikov V.L. Kubrina O.Yu. Oreshkina A.A. Features of the course of communityacquired pneumonia Vestnik Penzenskogo gosudarstvennogo universiteta 2019; 25(1):68–73.

[13] Chubukova O.A. Shkarin V.V. Features of the epidemiology of community-acquired pneumonia with combined etiology. Medicinskij al’manah 2017; 49(4):149–156.

[14] Zimenkov D.V. Kulagina E.V. Antonova O.V. Zhuravlev V.Y. Gryadunov D.A. Simultaneous drug resistance detection and genotyping of Mycobacterium tuberculosis using a low-density hydrogel microarray. J Antimicrob Chemother 2016; 71(6):1520–1531, 10.1093/jac/dkw015 26929267

[15] Fesenko E.E. Kireyev D.E. Gryadunov D.A. Mikhailovich V.M. Grebennikova T.V. L’vov D.K. Zasedatelev A.S. Oligonucleotide microchip for subtyping of influenza A virus. Influenza Other Respir Viruses 2007; 1(3):121–129, 10.1111/j.1750-2659.2007.00018.x 19453417 PMC4941880

[16] Shaik A.H. Govindan V. Nagraj G. Ravikumar K.L. Development of a microarray-based method for simultaneous detection and serotyping of Streptococcus pneumoniae from culture negative serum samples. J Appl Biol Biotech 2019; 7(5):15–24, 10.7324/jabb.2019.70503

[17] Leski T.A. Lin B. Malanoski A.P. Stenger D.A. Application of resequencing microarrays in microbial detection and characterization. Future Microbiol 2012; 7: 625–637, 10.2217/fmb.12.30 22568717

[18] Tokman H.B. Aslan M. Ortaköylü G. Algingil R.C. Yüksel P. Karakullukçu A. Kalayci F. Saribaş S. Cakan H. Demir T. Kocazeybek B.S. Microorganisms in respiratory tract of patients diagnosed with atypical pneumonia: results of a research based on the use of reverse transcription polymerase chain reaction (RT-PCR) DNA microarray method and enzymelinked immunosorbent assay. Clin Lab 2014; 60(6): 1027–1034, 10.7754/clin.lab.2013.130731 25016709

[19] Ma X. Li Y. Liang Y. Liu Y. Yu L. Li C. Liu Q. Chen L. Development of a DNA microarray assay for rapid detection of fifteen bacterial pathogens in pneumonia. BMC Microbiol 2020; 20(1): 177, 10.1186/s12866-020-01842-3 32576241 PMC7310556

[20] You Y.H. Wang P. Wang Y.H. Wang H.B. Yu D.Z. Hai R. Zhang J.Z. Assessment of comparative genomic hybridization experiment by an in situ synthesized Combi Matrix microarray with Yersinia pestis vaccine strain EV76 DNA. Biomed Environ Sci 2010; 23(5):384–390, 10.1016/s0895-3988(10)60080-3 21112487

[21] Filatova E.N. Chaikina A.S. Brusnigina N.F. Makhova M.A. Utkin O.V. An algorithm for the selection of probes for specific detection of human disease pathogens using the DNA microarray technology. Sovremennye tehnologii v medicine 2022; 14(1): 6, 10.17691/stm2022.14.1.01 PMC9376759 35992996

[22] National Center for Biotechnology Information. Nucleotide. URL: https://www.ncbi.nlm.nih.gov/nucleotide.

[23] Caruthers M.H. Gene synthesis machines: DNA chemistry and its uses. Science 1985; 230(4723):281–285, 10.1126/science.3863253 3863253

[24] CustomArray Inc. URL: http://www.customarrayinc.com.

[25] R Core Team. R: a language and environment for statistical computing. Vienna: R Foundation for Statistical Computing; 2014. URL: http://www.R-project.org/.

[26] Liu R.H. Dill K. Fuji H.S. McShea A. Integrated microfluidic biochips for DNA microarray analysis. Expert Rev Mol Diagn 2006; 6(2):253–261, 10.1586/14737159.6.2.253 16512784

[27] Camacho C. Coulouris G. Avagyan V. Ma N. Papadopoulos J. Bealer K. Madden Th.L. BLAST+: architecture and applications. BMC Bioinformatics 2009; 10: 421, 10.1186/1471-2105-10-421 20003500 PMC2803857

[28] Raman T. O’Connor T.P. Hackett N.R. Wang W. Harvey B.G. Attiyeh M.A. Dang D.T. Teater M. Crystal R.G. Quality control in microarray assessment of gene expression in human airway epithelium. BMC Genomics 2009; 10: 493, 10.1186/1471-2164-10-493 19852842 PMC2774870

[29] Kosikowska U. Biernasiuk A. Rybojad P. Łoś R. Malm A. Haemophilus parainfluenzae as a marker of the upper respiratory tract microbiota changes under the influence of preoperative prophylaxis with or without postoperative treatment in patients with lung cancer. BMC Microbiol 2016; 16: 62, 10.1186/s12866-016-0679-6 27052615 PMC4823876

[30] Pickering J. Richmond P.C. Kirkham L.A. Molecular tools for differentiation of non-typeable Haemophilus influenzae from Haemophilus haemolyticus. Front Microbiol 2014; 5: 664, 10.3389/fmicb.2014.00664 25520712 PMC4251515

[31] Lin B. Wang Z. Vora G.J. Thornton J.A. Schnur J.M. Thach D.C. Blaney K.M. Ligler A.G. Malanoski A.P. Santiago J. Walter E.A. Agan B.K. Metzgar D. Seto D. Daum L.T. Kruzelock R. Rowley R.K. Hanson E.H. Tibbetts C. Stenger D.A. Broad spectrum respiratory tract pathogen identification using resequencing DNA microarrays. Genome Res 2006; 16(4):527–535, 10.1101/gr.4337206 16481660 PMC1457032

[32] Spuesens E.B. Fraaij P.L. Visser E.G. Hoogenboezem T. Hop W.C. van Adrichem L.N. Weber F. Moll H.A. Broekman B. Berger M.Y. van Rijsoort-Vos T. van Belkum A. Schutten M. Pas S.D. Osterhaus A.D. Hartwig N.G. Vink C. van Rossum A.M. Carriage of Mycoplasma pneumoniae in the upper respiratory tract of symptomatic and asymptomatic children: an observational study. PLoS Med 2013; 10(5): e1001444, 10.1371/journal.pmed.1001444 23690754 PMC3653782

[33] Schmidt S.M. Müller C.E. Mahner B. Wiersbitzky S.K. Prevalence, rate of persistence and respiratory tract symptoms of Chlamydia pneumoniae infection in 1211 kindergarten and school age children. Pediatr Infect Dis J 2002; 21(8):758–762, 10.1097/00006454-200208000-00012 12192165

[34] Miyashita N. Niki Y. Nakajima M. Fukano H. Matsushima T. Prevalence of asymptomatic infection with Chlamydia pneumoniae in subjectively healthy adults. Chest 2001; 119(5):1416–1419, 10.1378/chest.119.5.1416 11348947

[35] Ramirez J.A. Ahkee S. Tolentino A. Miller R.D. Summersgill J.T. Diagnosis of Legionella pneumophila, Mycoplasma pneumoniae, or Chlamydia pneumoniae lower respiratory infection using the polymerase chain reaction on a single throat swab specimen. Diagn Microbiol Infect Dis 1996; 24(1):7–14, 10.1016/0732-8893(95)00254-5 8988757

[36] Ghindilis A.L. Smith M.W. Schwarzkopf K.R. Roth K.M. Peyvan K. Munro S.B. Lodes M.J. Stöver A.G. Bernards K. Dill K. McShea A. CombiMatrix oligonucleotide arrays: genotyping and gene expression assays employing electrochemical detection. Biosens Bioelectron 2007; 22(9–10): 1853–1860, 10.1016/j.bios.2006.06.024 16891109

